# Targeting FTO for cancer therapy and more

**DOI:** 10.18632/aging.203404

**Published:** 2021-08-11

**Authors:** Ying Qing, Rui Su, Jianjun Chen

**Affiliations:** 1 Department of Systems Biology, Beckman Research Institute of City of Hope, Monrovia, CA 91016, USA; 2 City of Hope Comprehensive Cancer Center, City of Hope, Duarte, CA, 91010, USA; 3 Gehr Family Center for Leukemia Research, City of Hope, Duarte, CA, 91010, USA

**Keywords:** acute myeloid leukemia, aging, cancer, epigenetics, experimental cancer therapeutics

Cancer is considered an age-related disease as the incidence rate of cancer rises with advancing age: in the United States, 80% of all cancer cases are diagnosed in the population aged 55 years and older [1]. Patients with advanced age tend to present with poorer performance status and have a worse prognosis for multiple types of cancer, including acute myeloid leukemia (AML), glioblastoma, breast cancer, and pancreatic cancer. Particularly, age is an independent prognostic factor in AML, and elderly patients are more vulnerable to treatment toxicities and have a much higher rate of developing multidrug resistance (57% - 62% for AML patients older than 56 years compared to 33% for AML patients younger than 56 years) [2]. Therefore, there is an urgent need for novel targeted therapeutics with minimal toxicity that can overcome drug resistance to improve the clinical outcomes in elderly cancer patients.

**Figure 1:**
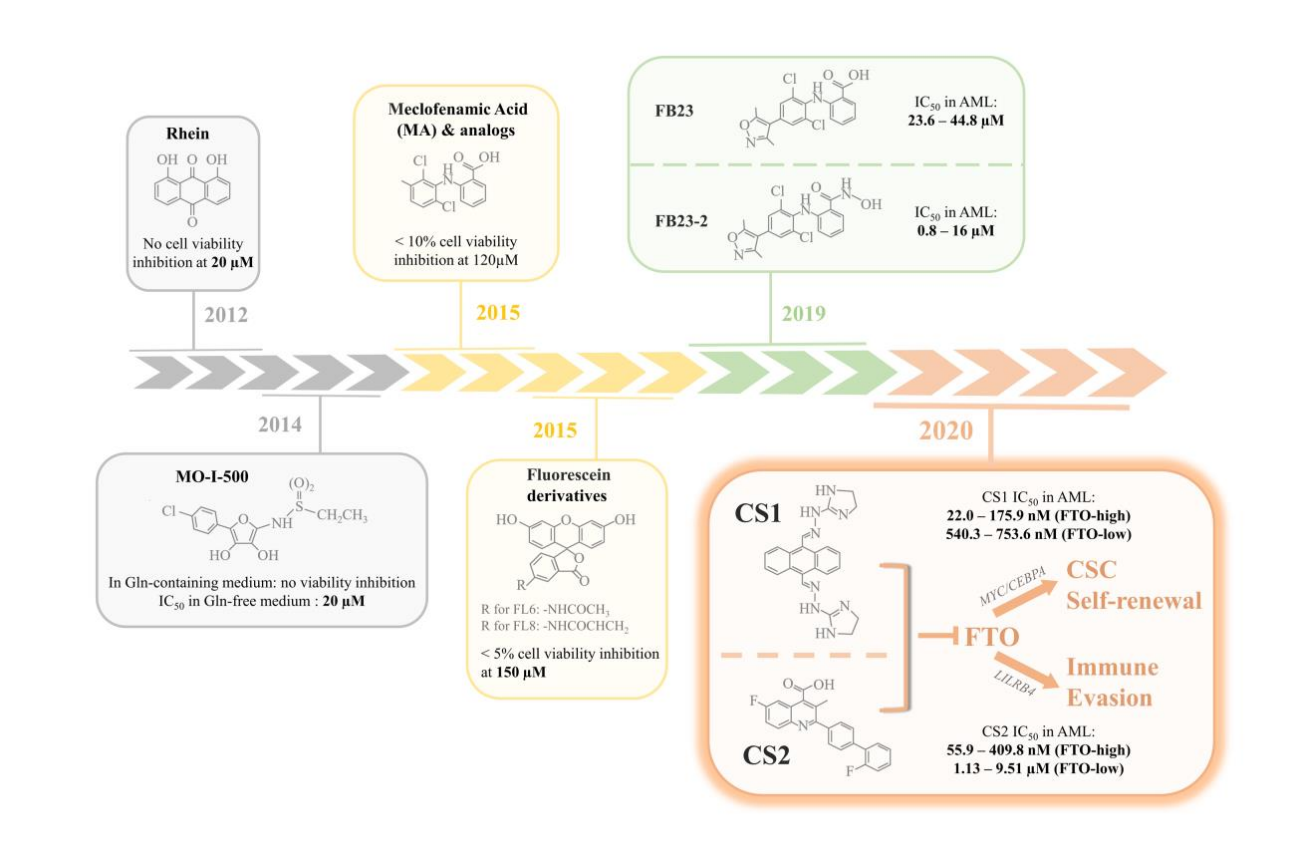
**Evolutional landmarks in FTO inhibitor discovery.** Rhein, the first FTO inhibitor identified in 2012, did not impair viability in BE(2)-C neuroblastoma cells at a dose of 20 &mu;M. MO-I-500, an inhibitor discovered in 2014, was found to inhibit survival of SUM149 breast cancer cells in glutamine (Gln)-free medium with an IC_50_ of 20 &mu;M, but had little effect on cells cultured in complete medium. Meclofenamic acid (MA) and its analogs were reported to be highly selective inhibitors of FTO. However, more than 90% of the Hela cells treated with 120 &mu;M MA2 (an MA analog) remained viable. Fluorescein and its derivatives could simultaneously inhibit and label FTO and were therefore considered "bifunctional". At a concentration of up to 150 &mu;M, fluorescein derivatives FL6 and FL8 did not display inhibitory effects on Hela cells with > 95% viable cells. In 2019, guided by the structural complex of FTO/MA, FB23 and FB23-2 were designed and optimized as two more potent FTO inhibitors. But the IC_50_ for both inhibitors in acute myeloid leukemia (AML) were still in the micromolar range (23.6 – 44.8 &mu;M for FB23 and 0.8 – 16 &mu;M for the optimized FB23-2). In contrast, the most recently discovered small molecule FTO inhibitors, CS1 and CS2, have much lower IC_50_ values in AML and solid tumors, especially in cancer cells that highly express FTO (in the low nanomolar range). CS1 and CS2 exert their anti-tumor activity by suppressing the FTO-mediated upregulation of *MYC*/*CEBPA* as well as *LILRB4*, thereby attenuating cancer stem cell (CSC) self-renewal and overcoming tumor immune evasion.

The epitranscriptional modification *N*^6^-Methyladenosine (m^6^A), the most abundant internal mark of eukaryotic mRNAs, plays critical roles in cancer development and is characterized by its reversibility and susceptibility to external regulation [3]. m^6^A is installed by "writers" (methyltransferases) and removed by "erasers" (demethylases), with m^6^ "reader" (m^6^A-binding proteins) responsible for recognizing the m^6^A-modified sites on target transcripts to mediate the downstream biological consequences. As the first identified m^6^A demethylase, FTO alpha-ketoglutarate dependent dioxygenase (*FTO*) has been reported to be highly expressed in certain AML subtypes, and promotes pro-survival signaling as well as blocks myeloid differentiation by targeting a set of genes such as *ASB2*, *RARA*, *MYC* and *CEBPA* in an m^6^A-dependent way [4,^5^]. Moreover, FTO positively regulates the glycolytic genes *PFKP* and *LDHB* to maintain aerobic glycolysis in leukemia cells [6]. *FTO* also serves as an oncogene in various solid tumors where it is aberrantly overexpressed, including glioblastoma, breast cancer, and pancreatic cancer [3]. The experimental evidences that knockdown of *FTO* effectively suppresses tumor progression, attenuates cancer cell metabolism, and improves the response of cancer cells to drug treatment strongly suggest FTO to be a promising therapeutic target for cancer treatment in elderly patients [4,^6^]. Based on these data, increased efforts have been made for the discovery of effective small-molecule FTO inhibitors. However, most previously discovered FTO inhibitors, although shown to be a potential therapeutic strategy for AML and solid tumors, only demonstrate mild/moderate efficacy and/or selectivity in inhibiting FTO (with their IC_50_ values higher than 1 &mu;M), and are therefore unlikely clinically applicable (Figure 1).

Most recently, Su et al. identified two highly efficacious small-molecule FTO inhibitors, i.e., CS1 (or Bisantrene) and CS2 (or Brequinar), which display potent *in vitro* and *in vivo* anti-tumor effects in both AML and solid tumors in which *FTO* is highly expressed (including glioblastoma, breast cancer, and pancreatic cancer) [7]. By blocking the catalytic pocket and disrupting the binding of FTO to m^6^A modified targets, CS1 and CS2 inhibit FTO activity and signaling. The IC_50_ values for both CS1 and CS2 are in the low nanomolar range, indicating that they are much more effective than previously discovered FTO inhibitors. Importantly, treatment with CS1 or CS2 induced minimal drug toxicity in C57BL/6 mice, even at a dose 4-fold higher than that used for cancer treatment. Since elderly cancer patients are generally less tolerant to chemotherapy and have a greater risk of drug toxicity, these FTO-targeting compounds provide a potential alternative treatment for the chemotherapy-intolerant elderly patients. Hypomethylating agents (HMAs) are also commonly used in frontline therapy for elderly AML patients unfit for intensive chemotherapy. However, most HMA-treated patients eventually develop drug resistance as a result of upregulation of immune checkpoint gene expression and subsequent immune evasion. CS1 and CS2 treatments sensitize AML cells to T cell cytotoxicity by suppressing the expression of immune checkpoint gene *LILRB4*, supporting FTO inhibition as an effective strategy to overcome immune evasion induced by HMAs (Figure 1). Pharmacological inhibition of FTO with the two compounds or genetic depletion of FTO also leads to a remarkable decrease in self-renewal capacity of the leukemia stem/initiating cells (LSCs/LICs), the major population considered to be responsible for treatment failure and disease relapse in AML (Figure 1). Hence, potent small-molecule FTO inhibitors (represented by CS1 and CS2) hold great therapeutic potential, alone or in combination with other agents such as HMAs, for the treatment of refractory and relapsed cancer in elderly patients.

The *FTO* gene is also the first genome-wide association study (GWAS)-identified locus that harbors the strongest genetic association with obesity. Although controversy exists, multiple lines of evidences support a positive relationship between *FTO* expression and the development of obesity [8]. With the continuous expansion of the obesity and overweight populations especially in the elderly, the effects of small-molecule FTO inhibitors on obesity development represent another major area of scientific inquiry to explore whether multiple health benefits (i.e. prevention of cancer and obesity/overweight) can be achieved concomitantly by targeting FTO.
